# A prolonged run-in period of standard subcutaneous microdialysis ameliorates quality of interstitial glucose signal in patients after major cardiac surgery

**DOI:** 10.1038/s41598-018-19768-2

**Published:** 2018-01-19

**Authors:** Othmar Moser, Julia Münzker, Stefan Korsatko, Christoph Pachler, Karlheinz Smolle, Wolfgang Toller, Thomas Augustin, Johannes Plank, Thomas R. Pieber, Julia K. Mader, Martin Ellmerer

**Affiliations:** 1 Division of Endocrinology and Diabetology, Department of Internal Medicine, Medical University of Graz, Graz, Austria; 2 Diabetes Research Group, School of Medicine, Swansea University, Swansea, United Kingdom; 3 Applied Sport, Technology, Exercise and Medicine Research Centre (A-STEM), College of Engineering, Swansea University, Swansea, United Kingdom; 4 Department of Cardiology and Intensive Care Medicine, LKH Graz Süd-West, Graz, Austria; 5 Intensive Care Unit, Department of Internal Medicine, Medical University of Graz, Graz, Austria; 6 Department of Anaesthesiology, Medical University of Graz, Graz, Austria; 7 Institute for Biomedicine and Health Sciences, Joanneum Research GmbH, HEALTH, Graz, Austria; 8 Division of Gastroenterology and Hepatology, Department of Internal Medicine, Medical University of Graz, Graz, Austria

## Abstract

We evaluated a standard subcutaneous microdialysis technique for glucose monitoring in two critically ill patient populations and tested whether a prolonged run-in period improves the quality of the interstitial glucose signal. 20 surgical patients after major cardiac surgery (APACHE II score: 10.1 ± 3.2) and 10 medical patients with severe sepsis (APACHE II score: 31.1 ± 4.3) were included in this investigation. A microdialysis catheter was inserted in the subcutaneous adipose tissue of the abdominal region. Interstitial fluid and arterial blood were sampled in hourly intervals to analyse glucose concentrations. Subcutaneous adipose tissue glucose was prospectively calibrated to reference arterial blood either at hour 1 or at hour 6. Median absolute relative difference of glucose (MARD), calibrated at hour 6 (6.2 (2.6; 12.4) %) versus hour 1 (9.9 (4.2; 17.9) %) after catheter insertion indicated a significant improvement in signal quality in patients after major cardiac surgery (p < 0.001). Prolonged run-in period revealed no significant improvement in patients with severe sepsis, but the number of extreme deviations from the blood plasma values could be reduced. Improved concurrence of glucose readings via a 6-hour run-in period could only be achieved in patients after major cardiac surgery.

## Introduction

In recent years, clinical management of glycaemia in critically ill patients has tremendously changed. Glucose levels within a low- normal range were shown to be associated with prolonged hospital stay and increased risk of death^1^. In addition to life-threatening low glucose levels and hypoglycaemia, hyperglycaemia was found to be associated with a rise in morbidity due to infections and higher incidence of post-surgical infections^2^. Intriguingly, mild hyperglycaemia with 7.8–10 mmol/l reduced both the rate of morbidity and mortality in a greater extent than strict euglycaemia (4.5–6 mmol/l) in different groups of critically ill patients. Evidence suggests that near-normal target ranges (7.2 – 8 mmol/l) are safe and do also translate to lower mortality. To achieve these goals, glucose concentration should be measured by repeated arterial measurements using a blood gas analyser - the gold standard in critical care^3^. Regular blood glucose testing on the other hand is associated with increased workload for nursing staff and might reduce adherence to established protocols aiming for euglycaemia and reduced glucose variability^4^.

Alternative methods for glucose determination which decrease the workload for nursing staff could overcome these issues. Continuous subcutaneous glucose monitoring either by using commercially available continuous monitoring (CGM) systems^5–9^ or by means of standard microdialysis^10–13^ has been proven to accurately track changes in glucose concentration in both patients with type 1 and type 2 diabetes mellitus. Not only acute also long long-term clinical outcomes were found to be improved when using CGM systems. In a recent analysis within the T1D Exchange clinic registry, glycaemic control was significantly ameliorated in patients using CGM than those who did self-monitoring of blood glucose (SMBG), regardless of using an insulin pump or multiple daily injections via insulin pen^14^.

One of the remaining challenges in CGM is system calibration which should be performed at an optimal time point (i.e. during stable glycaemia) to allow for sufficient accuracy of the CGM signal. A reliable CGM system could help monitoring changes in glycaemia in a higher resolution, thus preventing life-threatening hypo- and hyperglycaemic episodes.

Venous microdialysis was shown to be a highly accurate and reliable method for continuous blood glucose monitoring up to 48 hours in patients undergoing cardiac surgery in intensive care units (ICU)^15^, but if the subcutaneous tissue is also a reliable spot for glucose measurement has not been finally proven. In patients undergoing cardiac surgery it was found that subcutaneous CGM systems repeatedly underestimated glucose values, which was not supposed to be linked to impaired microcirculation^8^. In septic patients, Kopterides *et al*. recommended microdialysis measurement only for research purposes due to insufficient studies in this patient population^16^. Additionally, it is known that microdialysis^16^ as well as CGM systems^17^ differ in relation to the stage and type of sepsis, which might exacerbate obtaining a precise interstitial glucose measurement.

Fundamentally, a comparison of surgical and medical patients is of interest as both groups differ in various aspects that may alter interstitial glucose sensor performance (e.g. catheter-related bloodstream infections, alterations in microvascular blood flow)^18,19^. Recommendations for glucose treatment in ICU patients are not specified for surgical and medical patients^2^ and in addition it is still not yet investigated whether different types of critical illnesses have different effects on the accuracy of a microdialysis-based CGM signal. Furthermore, it needs to be proven if the time point of calibration influences the quality of the measurement compared to reference values. The aim of the present study was to investigate the accuracy of continuous subcutaneous glucose monitoring by means of microdialysis in two different critically ill patient populations (surgical patients after major cardiac surgery (SICU group) and medical patients with severe sepsis (MICU group) and to investigate whether the prolongation of the run-in period improves the signal quality. As a working hypothesis, we expected stable perfusion of the probe after introduction of a 6-hour run-in period, reflected by an improvement in the subcutaneous glucose signal in both patient populations.

## Methods

The current study comprises data from two trials (14–189 ex 03/04; 16–233 ex 04/05), which were performed in two populations including overall 30 critically ill patients (Table 1): 20 patients at the surgical intensive care unit after major cardiac surgery (SICU group) and 10 patients at the medical intensive care unit with severe sepsis (MICU group). The SICU group was treated at the Department of Cardiothoracic Surgery and MICU group was treated at the Department of Internal Medicine, both at the Medical University of Graz, Austria. These studies were mono-centric, non-controlled prospective observational experiments. The study protocols were approved before the year 2010 by the local ethics committee of the Medical University of Graz, Austria, and therefore not registered in a clinical trial registry. Both studies were performed according to Good Clinical Practice (GCP) and the Declaration of Helsinki. Patients were treated according to the best practice and no other study related intervention was performed. Nutrition management was performed according to local standards. Patients received enteral nutrition via tube with a flow rate of 50–80 ml/h from 8:00 AM to 6:00 AM the following day. Parenteral nutrition was administered continuously aiming at 20–25 kcal/kg bodyweight; septic patients received 50% more than patients without sepsis. In the MICU group, patients were under mechanical ventilation; written informed consent was obtained from the closest relative before starting study related activities. SICU patients gave their written informed consent before the elective cardio-thoracic surgery and prior to any trial related activities.

**Table 1:** Subject characteristics. BMI = body mass index, SICU = surgical patients after major cardiac surgery, MICU = medical patients with severe sepsis. Values are given as mean and SD. APACHE II score = Acute physiology and chronic health evaluation.

	SICU (n = 20)	MICU (n = 10)
Age (years)	69 ± 7	57 ± 11
Ethnicity (Caucasian)	20	10
Female gender (n)	5 (25%)	5 (50%)
BMI (kg/m^2^)	28 ± 5	35 ± 15
History of diabetes (n)	6 (30%)	2 (20%)
APACHE II score	10 ± 3	31 ± 4

### Preparation

In all 30 patients, standard subcutaneous microdialysis for glucose measurement was applied. The sampling period was up to 24 hours in the MICU group and up to 48 hours in the SICU group. In both patient groups, a CMA 60 (CMA Microdialysis AB, Solna, Sweden) microdialysis probe (membrane cut-off: 20 kDa, 30 × 0.6 mm) was inserted into the subcutaneous adipose tissue of the abdominal region. Using a portable micropump (CMA 107; CMA Microdialysis AB, Solna, Sweden), the isotonic perfusion solution (5% mannitol) was transported through the catheter at a constant flow rate of 1 µl/min. Dialysate samples were collected in microtubes and tubes were changed every 60 min over the whole study period. A graphical illustration of the microdialysis principle is depicted in the supplemental material. Simultaneously, arterial blood samples were withdrawn to measure blood glucose reference concentrations every 60 min. Dialysate samples were immediately deep frozen (−80 °C) and later analysed for glucose concentrations using a Cobas Mira Analyzer (Hoffmann-La Roche, Basel, Switzerland) and for sodium concentrations using a flame photometer (Instrumentation Laboratory, Vienna, Austria) at the laboratory of Joanneum Research GmbH, Graz, Austria. Reference blood glucose concentrations were measured immediately at site using a blood gas analyser (Omni S, Roche Diagnostics, Basel, Switzerland). Blood glucose reference readings were used for prospectively calibrating the microdialysis signal. Intrarun (interrun) coefficients of variation were 1.3% (1.6%) and 0.4% (0.4%) for glucose and sodium, respectively.

The measured glucose concentration in subcutaneous dialysate was calibrated via two- steps: firstly, using an ionic reference technique to calculate actual subcutaneous glucose concentration and secondly, using a one-point calibration to obtain a glucose profile comparable to arterial blood glucose^4^. The ionic reference calibration accounts for a lower measured dialysate glucose concentration in relation to the actual subcutaneous glucose concentration and is calculated for each sample using the following equation: actual subcutaneous glucose concentration = dialysate glucose x plasma sodium/dialysate sodium. As both plasma and tissue sodium concentrations can be considered relatively constant, fluctuations in dialysate sodium can be attributed mainly to effects at the site of the microdialysis membrane (SIUC dialysate sodium: 103.6 ± 17.7 mmol/l, SICU plasma sodium: 141.2 ± 2.93 mmol/l; MICU dialysate sodium: 111.9 ± 23.0 mmol/l, MICU plasma sodium: 139.1 ± 6.34 mmol/l). Actual subcutaneous glucose concentration, as calculated using the ionic reference technique, is lower compared with the actual arterial blood glucose concentration. Therefore, as a second calibration step, actual subcutaneous glucose concentration was calibrated to blood glucose using a one-point calibration procedure. Using this procedure, the ratio between the first actual subcutaneous glucose concentration reading and the average of the two corresponding blood glucose readings was used to calculate actual subcutaneous glucose concentration-derived blood glucose.

The calibration procedure was performed as previously described^4^ using the ionic reference technique to account for sodium recovery and then prospectively calibrating the subcutaneous signal to blood concentrations. Calibration was conducted as a one-point calibration either one or six hours after insertion of the microdialysis catheter. All laboratory analyses were performed according to Good Laboratory Practice (GLP).

### Data analysis

Data were tested for distribution via D’Agostino & Pearson omnibus normality test. Normally distributed data were expressed as mean ± standard deviation (SD) and non-normally distributed data were expressed as median and 25–75 percentiles. Comparison of standard subcutaneous microdialysis for glucose measurement with the corresponding arterial glucose concentration was performed via Wilcoxon matched-pairs signed rank test with a significance level of p < 0.05 for both groups. Paired reference – microdialysis values were used to calculate median difference, median absolute difference (MAD) and median absolute relative difference (MARD). MARD for the comparison of calibration 1 hour versus 6 hours after catheter insertion was also performed by Wilcoxon matched-pairs signed rank test with a significance level of p < 0.05 for both groups.

Subcutaneous microdialysis samples were collected over a predefined time period of 60 minutes. To account for the long sampling interval of one hour, the reference blood glucose measurements corresponding to a start and an end glucose value of the interstitial samples were averaged and then paired with the according interstitial glucose value, as described previously^20^. Bland-Altman plot was used to indicate the relative differences between the paired glucose readings on the y-axis in relation to the average blood glucose concentration on the x-axis^21,22^.

The Clarke Error Grid analysis^23^ was applied to clinically evaluate the data. Five zones characterise errors of varying levels of clinical significance, among which values in zones A and B are defined as clinically acceptable, whereas values in zones C, D or E are considered as potentially unsafe. The insulin titration error grid was used as described previously by Ellmerer *et al*.^4^.

Analyses were performed using SPSS 19 for Windows (SPSS, Chicago, IL, USA) and Microsoft Office Excel 2010.

### Data availability

The datasets generated during and/or analysed during the current study are available from the corresponding author on reasonable request.

## Results

Thirty critically ill patients were included in the analysis. Mean study duration was 37 hours in the SICU group and 26 hours in the MICU group.

In total, we obtained 596 paired glucose readings from the SICU group and 220 from the MICU group. For the SICU group, calibrating the microdialysis signal 1 hour after catheter insertion resulted in a prospectively calibrated glucose concentration of 6.7 (5.7; 8.3) mmol/l versus a reference value of 6.3 (5.5; 7.8) mmol/l (p < 0.001). For the MICU group, 1 hour post-catheter insertion calibrated subcutaneous glucose concentration was found at 8.2 (7.1; 9.9) mmol/l versus a reference value of 7.9 (6.9; 9.3) mmol/l (p = 0.014).

When accounting for a 6-hour run-in period, prospectively calibrated glucose concentration was found at 5.9 (5.2; 7.5) mmol/l in comparison to a reference arterial blood value of 6.1 (5.4; 7.5) mmol/l for the SICU group (p = 0.012). For the MICU group, glucose concentration calibrated at hour 6 was found at 8.1 (6.5; 10.0) mmol/l versus a reference arterial blood value of 7.9 (7.0; 9.3) mmol/l (p = 0.014).

Recovery rates for interstitial glucose (73 ± 10% SICU_h1_, 74 ± 10% SICU_h6_, 82 ± 12 MICU_h1_, 82 ± 12 MICU_h6_) and CV of recovery (1.5% SICU_h1_, 1.4% SICU_h6_, 3.4% MICU_h1_, 3.4% MICU_h6_) were comparable between groups.

Overall levels for median difference, MAD and MARD for both calibration time-points are given in Table 2. When comparing MARD of the glucose signal calibrated at 1 hour versus 6 hours after catheter insertion, a significant improvement in signal quality was found in patients after major cardiac surgery (p < 0.001). In patients with severe sepsis, introduction of a 6-hours run-in period after catheter insertion did not result in a significant improvement in the sensor quality assessed by MARD (p = 0.11).

**Table 2:** Median difference (MedDiff), median absolute difference (MAD) and median absolute relative difference (MARD) for group MICU and SICU calibrated 1 hour (MICU_h1_ and SICU_h1_) and 6 hours (MICU_h6_ and SICU_h6_) after catheter insertion. Values are given as median and 25–75 percentiles.

	SICU_h1_	MICU_h1_	SICU_h6_	MICU_h6_
MedDiff (mmol/l)	0.33 (−0.11; 0.87)	0.11 (−0.57; 1.09)	−0.06 (−0.44; 0.33)	2.89 (−0.59; 1.37)
MAD (mmol/l)	0.61 (0.27; 1.22)	0.88 (0.38; 1.75)	0.38 (0.16; 0.84)	0.88 (0.35; 1.83)
MARD (%)	9.9 (4.2; 17.9)	11.8 (5.0; 22.3)	6.2 (2.6; 12.4)	11.1 (4.8; 22.9)

**Figure 1:**
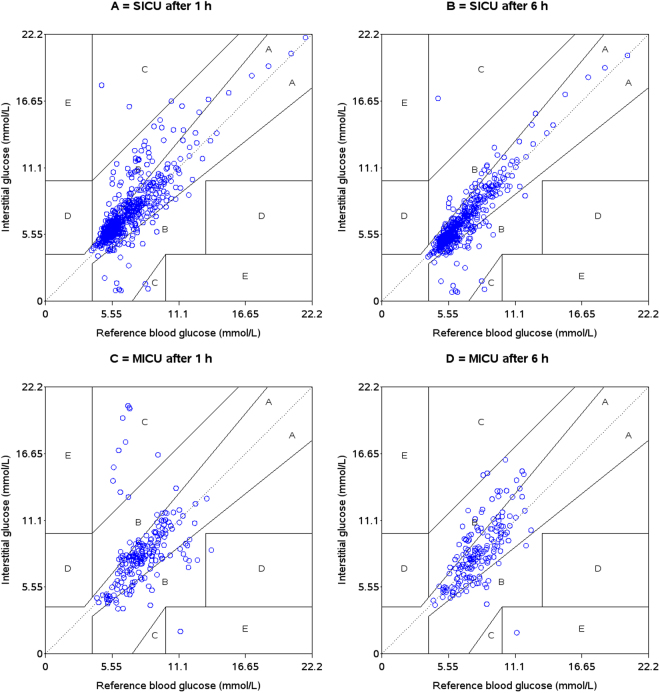
Mean glucose differences (2 SD) between interstitial and reference blood glucose concentrations. A = SICU calibrated after 1 h, B = SICU calibrated after 6 h, C = MICU calibrated after 1 h and D = MICU calibrated after 6 h.

The mean glucose differences (2 SD) between subcutaneous and reference glucose was 6.8 (47.8) % for SICU group and −18.8 (128.2) % for MICU group when calibrated 1 hour after catheter insertion. When calibrated at hour 6 after catheter insertion 2 SD were found at −2.2 (39.3) % (SICU group) and −7.2 (65.6) % (MICU group) (Fig. 1).

**Figure 2:**
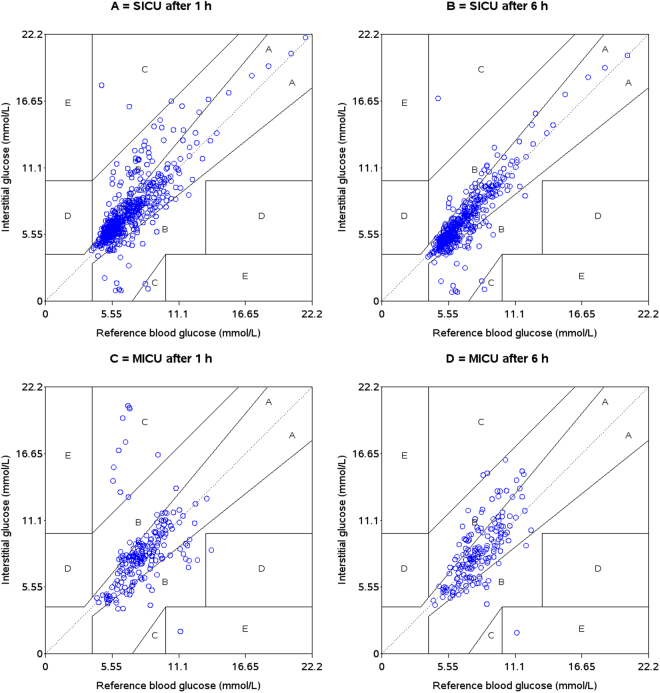
Clarke Error Grid analysis for A = SICU calibration 1 hour after catheter insertion, B = SICU calibration 6 hours after catheter insertion, C = MICU calibration 1 hour after catheter insertion, D = SICU calibration 6 hours after catheter insertion.

Clarke Error Grid analysis showed that 99.3% of the glucose values for SICU calibrated 1 hour after catheter insertion were in zones A and B (80.4% A, 18.9% B), whereas 4 values (0.7%) were located in zone C. For the MICU group, the glucose signal calibrated 1 hour after catheter insertion gave following results: 89.1% of the values were within zones A and B (71.4% A, 17.7% B), 9.9% were located in zone C, and one value was located in zones D and E, respectively. Calibration at hour 6 showed the following results for SICU: 87.9% in zone A, 11.7% in zone B and 0.4% in zone C; for MICU: 72.4% in zone A, 26.4% in zone B, 0.6% in zone C and 0.6% in zone E, respectively (Fig. 2).

## Discussion

CGM data from critically ill patients are scarce and clearly differ from results observed in patients with diabetes due to altered tissue perfusion^24^.

In a recent prospective study in critically ill patients assessing the performance of a continuous intravenous microdialysis-based glucose-monitoring device, trend accuracy was found to be very good. In contrast, point accuracy was only moderate to good, translating to neglect a threshold of 95% of paired values in zone A of the Clarke error grid^25^, which is similar to the findings in our study. In a different setting during surgery in ICU patients, a disagreement between subcutaneous and intravenous continuous glucose monitoring was found^26^. In this study the mean venous glucose level was 125 ± 29 mg/dl versus a subcutaneous glucose level of 138 ± 26 mg/dl^26^. Additionally, it has to be taken into account that the sensor function was found in previous research to be impaired by blood clots close to the sensor application site^27^. Blood injections, proximal to the sensor, caused lowered sensor glucose values and temporary signal reductions.

Contrary to the findings of Lorencio *et al*.^17^ our findings demonstrate considerable influence of the type of critically ill patient (medical or surgical) on the accuracy of the subcutaneous signal. Especially in patients with sepsis, the disease *per se* reduces the potential of the CGM device to measure glucose accurately. Both alterations in microvascular blood flow and decrease in density of small vessels were found to be present in patients with sepsis^19^. Further, different inflammatory mediators potentially decrease microvascular blood flow and increased endothelin leads to exaggerated microvascular vasoconstriction^28^. As stated by Martinez and colleagues^29^, metabolism in patients with sepsis differ markedly from that associated with circulatory failure, which might influence sensor accuracy.

In patients after cardiac surgery, it was found that CGM sensors had acceptable accuracy^30^. The microcirculation was impaired to a limited extent compared with that in patients with sepsis and healthy controls, although this impairment was not related to sensor accuracy. These findings are in line with our results from the SICU group, in which superior accuracy was observed compared to the MICU group, and prolongation of the run-in period up to 6 hours post-catheter insertion resulted in an improved sensor signal.

Although an offset was found when comparing subcutaneous and arterial blood glucose values, the subcutaneous signal could follow trends in blood glucose adequately in both groups. MARD for calibration at hour 1 was in both groups similar to microdialysis results seen in a previous study in patients with diabetes and could even outperform these results when introducing a 6-h run-in period in the SICU but not in the MICU group^20^. Clarke Error Grid Analysis also supports these findings: for SICU there was an increase in the number of values laying in zone A after introduction of the 6 hours run-in period (80.4 vs. 87.9%). Although there was also a trend of improved performance for the MICU group after introduction of the 6-hour run-in period, this result was only seen in zone B (17.7% vs. 26.4%), whereas numbers in the clinical accurate zone A did not change (71.4% vs. 72.4%). These results from the SICU group are similar to data observed in patients with diabetes^20^. In contrast to vascular microdialysis in critically ill patients, performance of subcutaneous microdialysis was worse and could also not reach comparable accuracy after introduction of a 6 hours run-in period^15^. Interestingly, our data, in line with previously published data on vascular microdialysis^15^, are close to the MARD of 10%, which is requested in diabetes care to enable non-adjunct continuous subcutaneous glucose monitoring for insulin dosing decisions and also fulfils criteria proposed for critical care (MARD < 14%)^31^.

A recent study clearly demonstrated the agreement of a point-of-care device when compared to laboratory assays in critically ill patients^32^. Bias, limits of agreement and coefficients of correlation were found for glucose at −0.8 (−1.4 to −0.2 g/dl, r = 0.985), which is below CLIA cut-off values. The accuracy of the point-of-care devices, as used in our study, supports the accuracy to initiate therapy without waiting for central laboratory measurements. However, we used the ionic reference technique for micro-dialysis recovery rates-correction which is accompanied with electrolyte-free perfusate. These sodium concentrations were analysed by a flame photometer (sample by sample); Unfortunately, real-time monitoring was not possible using this setting. However, further developments of this technique do allow real-time monitoring of the dialysates by infrared spectrometry. Vahlsing *et al*. used either acetate or mannitol as recovery marker added to the perfusate and marker losses, as determined by infrared spectrometry^33,34^.

When interpreting the data, it has to be taken into account that the results provided are based on two individual study protocols using an identical study design but a different sample size (n = 10 for MICU vs. n = 20 for SICU). As the aim of this comparison was to investigate whether different clinical conditions (septic state vs. elective cardiothoracic surgery) influence the subcutaneous signal, anthropometric parameters such as body composition (body mass index, BMI) do not match. There might be a different pattern of adipose tissue blood flow between lean and obese patients, which could also affect signal accuracy. Furthermore, this study is limited by its small patient cohort and the lack of a healthy control group.

Further studies need to investigate the accuracy of vascular and subcutaneous glucose monitoring systems in different groups of critically ill patients, leading to a deeper understanding of illness-dependent effects on the glucose monitoring. Furthermore, the effects of different illness states and body constitutions on CGM accuracy are not yet sufficiently investigated in critically ill patients.

## Conclusions

This study investigated the accuracy of a standard subcutaneous microdialysis technique for glucose monitoring in critically ill surgical patients after major cardiac surgery and in medical patients with severe sepsis. We conclude that the prolongation of a run-in period up to 6 hours results in an improvement of subcutaneous signal accuracy in patients after major cardiac surgery, but not in patients with severe sepsis, possibly due to altered tissue perfusion in the septic state. From a clinical point of view, it must be taken into account that standard subcutaneous microdialysis technique for glucose monitoring might not be adequate to obtain accurate glucose profiles in patients with severe sepsis. This inaccuracy cannot be ameliorated by a prolongation of the run-in period.

## Electronic supplementary material

Supplementary Figure S1
